# Characterization of a *Clostridioides difficile* outbreak caused by PCR ribotype 046, associated with increased mortality

**DOI:** 10.1080/22221751.2022.2049981

**Published:** 2022-03-21

**Authors:** Cecilia Magnusson, Sara Mernelius, Malin Bengnér, Torbjörn Norén, Lena Serrander, Sophie Forshell, Andreas Matussek

**Affiliations:** a Department of Infectious Diseases, Region Jönköping County, Jönköping and Department of Biomedical and Clinical Sciences, Linköping University, Linköping, Sweden; b Laboratory Medicine, Region Jönköping County, Jönköping and Department of Biomedical and Clinical Sciences, Linköping University, Linköping, Sweden; c Office for control of Communicable Diseases, Region Jönköping County, Jönköping, Sweden; d Faculty of Medicine and Health, Department of Laboratory Medicine, National Reference Laboratory for Clostridioides difficile, Clinical Microbiology, Örebro University, Örebro, Sweden; e Division of Clinical Microbiology, Department of Clinical and Experimental Medicine, Faculty of Health Sciences, Linköping University, Linköping, Sweden; f Division of Laboratory Medicine, Institute of Clinical Medicine, University of Oslo, Oslo, Norway; g Division of Laboratory Medicine, Oslo University Hospital, Oslo, Norway

**Keywords:** *Clostridioides difficile*, CDI, mortality, outbreak, epidemiology, ribotyping, whole-genome sequencing

## Abstract

This study describes a large nosocomial outbreak of *Clostridioides difficile* infections (CDI) dominated by ribotype (RT) 046 in a Swedish hospital. The present study aimed to examine the pathogenicity of this RT, explore epidemiological links by whole genome sequencing (WGS), and evaluate different interventions implemented to stop the outbreak. Clinical isolates (*n* =  366) collected during and after the outbreak were ribotyped and 246 isolates were subjected to WGS. Medical records of patients infected with the seven most common RTs were evaluated. RT046 was spread effectively throughout the hospital and was the most common among the 44 different RTs found (114/366 isolates). Infection with RT046 was associated with higher mortality compared to other strains (20.2% to 7.8%), although there were no differences in concomitant disease, age or antibiotic treatment. To control the outbreak, several measures were successfully implemented.

## Introduction

*Clostridioides difficile*, previously named *Clostridium difficile*, is a major cause of nosocomial diarrhoea and pseudomembranous colitis [1]. Some *C. difficile* ribotypes (RT) have been described as hypervirulent and especially RT027 (B1/NAP1) has been associated with large outbreaks with severe outcomes since 2001 [2,3]. The incidence of RT027 is now decreasing, but instead, an increase in the prevalence of other virulent RTs, such as 017 and 078, associated with multiple antimicrobial resistance, has been seen globally [4,5,6,7].

In April 2011, an insidious outbreak of *C. difficile* infection (CDI) was discovered at Högland Hospital, Region Jönköping County, Sweden. The outbreak was recognized through a national surveillance program and local reports of increased incidence of CDI. It was mainly caused by a multi-drug resistant *C. difficile* of RT046, previously rarely detected in the region [8]. The Swedish Public Health Agency reported in their national surveillance of *C. difficile* 2008–2009 that RT046 was found in Region Jönköping County repeatedly, but only rarely in other Swedish counties [9]. In a European survey from 2008, the prevalence of RT046 was 2% [10]. Another European survey from 2014 found that RT046 was common in Italy [5]. RT046 has also been isolated in China and has been described in two minor outbreak situations [4,11,12].

This study aimed to examine the pathogenicity of RT046 compared to other RTs. Furthermore, we evaluated the course of the outbreak and the interventions implemented as well as the molecular epidemiology of the outbreak through ribotyping and whole-genome sequencing (WGS).

## Material and methods

### Setting and outbreak overview

**Figure 1.**
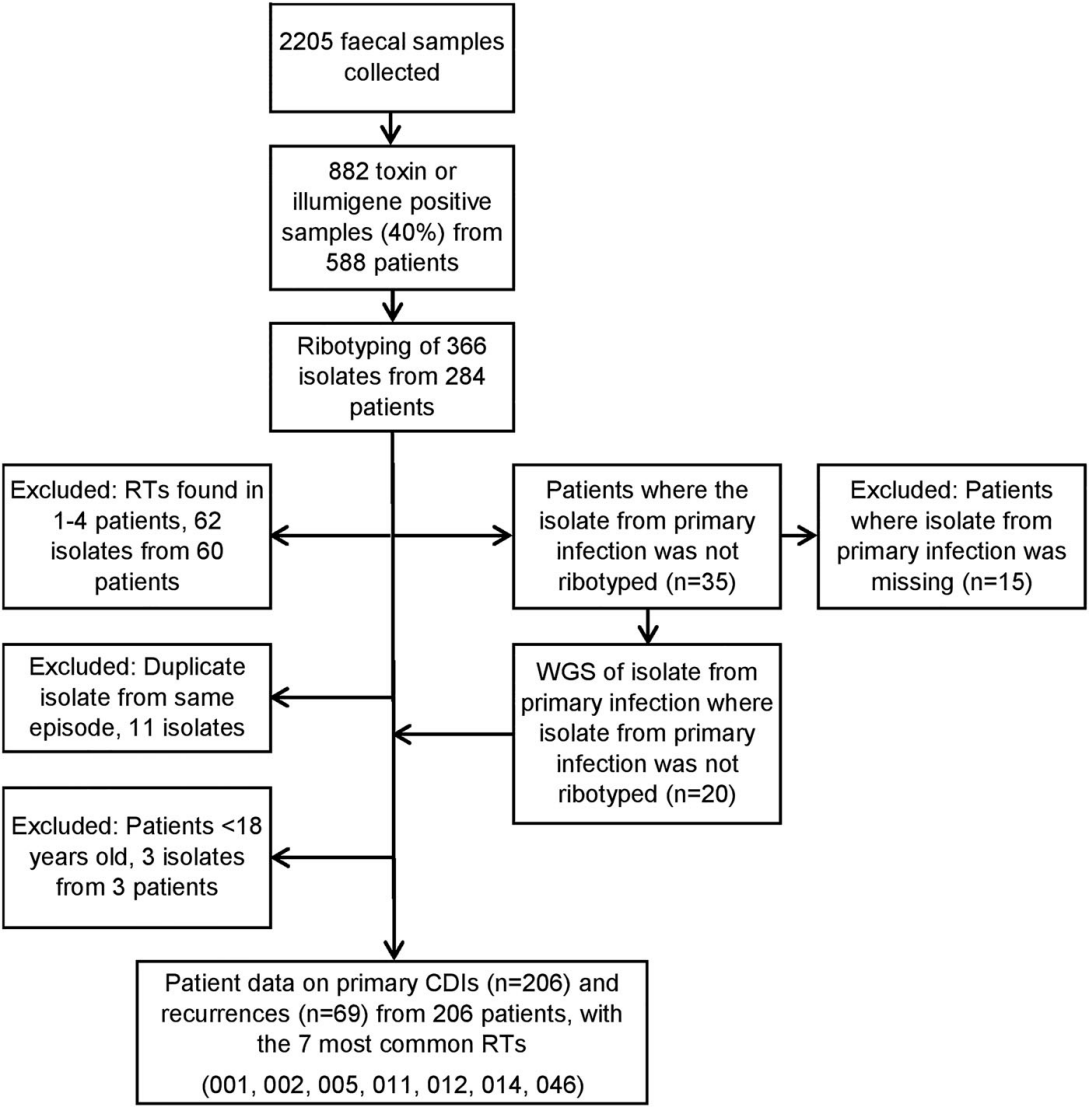
Flowchart of isolates included in the study from Högland Hospital catchment area, 2010–2011.

Högland Hospital, is a general hospital in Region Jönköping County, Sweden, with 248 beds at the time of the outbreak. CDI was one of the most common nosocomial infections in the hospital when the outbreak was discovered, and the average incidence (July–Dec 2011) was 22 cases compared to 10 and 7 cases per 10,000 hospital days in the other two hospitals in the county. In the catchment area of Högland Hospital, 433 samples, from 298 patients, were positive for *C. difficile* in routine diagnostic analyses in 2010 and 449 samples, from 290 patients, were positive in 2011. A collection of clinical isolates (*n* = 366) from 2010 and 2011 were subjected to ribotyping (Figure 1).

**Figure 2.**
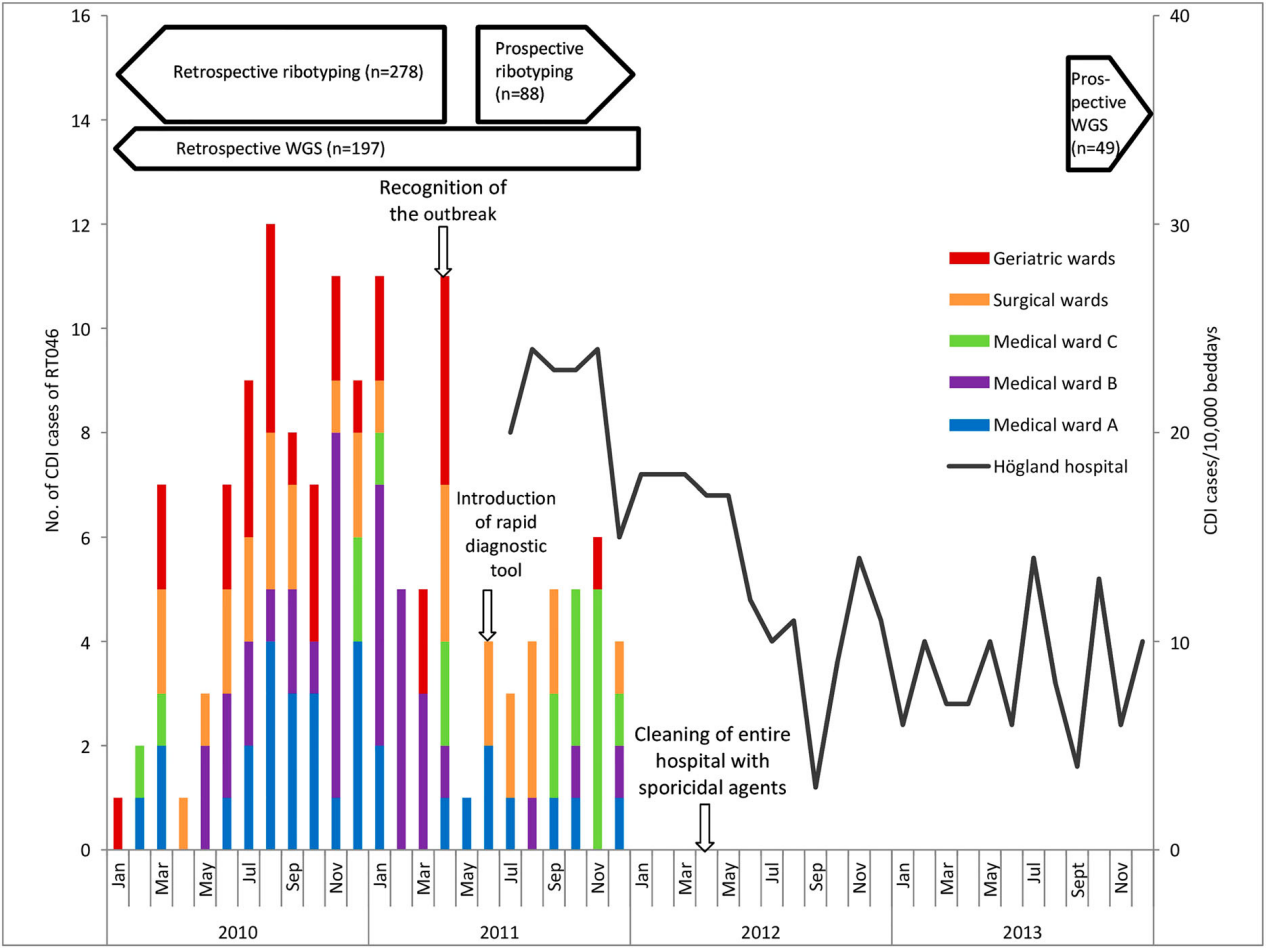
Timeline for CDI outbreak and interventions at Högland Hospital. Epidemiological curve showing CDI cases caused by RT046 at different wards at Högland Hospital during the outbreak (2010–2011) and CDI cases/10,000 bed days (July 2011–2013).

To contain the outbreak, an outbreak team was established in April 2011, initiating infection control measures. These included monthly evaluations of new CDIs, reinforcement of adherence to standard precautions and existing cleaning procedures, implementation of a rapid molecular diagnostic tool as a 24/7 service (Illumigene™ C. difficile, MeridianBioscience Inc., Cincinnati, OH) and antibiotic stewardship [13,14,15]. The antibiotic stewardship consisted of reintroduction and enlightenment of existing antibiotic guidelines to all prescribers at Högland Hospital. Also, the use of third-generation cefalosporines was evaluated monthly and reported back to prescribers. Due to the persistence of the outbreak, a second intervention was launched in April 2012 when the entire hospital was thoroughly cleaned using sporicidal 0.1% chlorine agent. During cleaning, the wards were temporarily emptied of patients [15]. For a schematic overview of the outbreak and interventions made to contain the outbreak, see Figure 2.

### Definitions

CDI was defined as ≥3 episodes of diarrhoea/24 h and a stool sample positive for *C. difficile*. An episode was defined as primary if the patient had no history of CDI within the last 8 weeks; otherwise, it was defined as a recurrence [16,17].

A primary episode was defined as hospital associated (HA) if it started ≥48 h after hospital admission or up to 4 weeks after discharge from hospital. A primary episode that started ≥12 weeks after discharge or if the patient did not have a history of hospitalization was defined as community associated (CA). If a primary episode started in 4–12 weeks after discharge it was defined as indeterminate [16,17].

Patients who had received at least one dose of carbapenemes, quinolones, clindamycin, or cephalosporins within 3 months before their CDI were considered exposed to CDI high-risk antimicrobials [18].

One-year overall mortality was recorded for patients after their primary infection. If death from any cause occurred within 30 days after the primary infection, the CDI was considered to have contributed to the cause of death.

### Review of medical records

Parameters collected from the medical records of patients ≥18 years were HA/CA CDI, recurrent infection, length of hospital stay, admissions to different wards before and at primary/recurrent infection, intensive care unit (ICU) status and 1-year overall mortality after CDI. Antibiotic treatment before CDI, proton pump inhibitor (PPI) treatment, CDI treatment medications, comorbidities scored by Charlson comorbidity index [19], and laboratory findings, including blood leukocytes count, creatinine, lactate, and C-reactive protein (CRP) were also collected.

If an RT was only found in sporadic cases, it was not considered prone to transmit, and therefore clinical characteristics were not collected for these patients (Figure 1).

Clinical characteristics were compared for patients with primary CDI caused by RT046 (*n* = 104) and patients with the subsequent six most common RTs (*n* = 102). The latter group is referred to as the comparison group.

### Diagnostic tests and culture

*C. difficile* was identified using the routine method put in place in the laboratory, which initially was the detection of TcdA and TcdB on faecal specimens using an enzyme-linked ﬂuorescent immunoassay (ELFA, Vidas CDAB assay, bioMerieux, Marcy-l’Étoile, France) and from June 2011 by Illumigene™ *C. difficile* (MeridianBioscience Inc., Cincinnati, OH), both performed according to the manufacturers’ instructions. All *C. difficile* positive specimens were cultured anaerobically on cycloserine-cefoxitin-fructose-agar for 48 h and retained *C. difficile* isolates were stored at −80°C.

### Isolates selected for ribotyping and whole-genome sequencing

Ribotyping was performed on 366 clinical isolates. Of these, 278 were a selection of stored isolates collected from January 2010 through May 2011, to investigate if there was a dominance of any RT. The remaining 88 isolates, collected from June to December 2011, were prospectively ribotyped to evaluate Illumigene as a diagnostic method. As of January 2012, no more ribotyping was performed. To further investigate the molecular epidemiology of the outbreak, WGS was performed on a selection of 177 isolates collected during January 2010 to December 2011. After the outbreak, WGS was performed on 49 consecutive isolates, collected during October 2013 through June 2014, from unique individuals to analyse if the outbreak strain still circulated. Additional 20 isolates from primary infections were analysed using WGS to establish the, previously unknown, ribotype in cases where only the type of the recurrent infection was known (Figures 1 and 2).

### PCR ribotyping

PCR ribotyping was performed, at the Swedish National Reference Laboratory for *Clostridioides difficile* as described elsewhere [20]. The internationally accepted nomenclature (ECDC-Cardiff collection) was applied for classifying RTs [21]. Swedish nomenclature was used for types not available in the international register. A reference RT046 strain was included to validate the outbreak RT strains.

### Whole-genome sequencing

WGS was performed on 246 isolates. *C. difficile* isolates were grown anaerobically and genomic DNA was extracted after lysing the bacteria, using the EZ1 DNA Tissue kit (Qiagen, Hilden, Germany). WGS was performed at the SciLifeLab (Solna, Sweden). One µl of normalized genomic DNA (1.5 ng/µl) was used in the tagmentation reaction using Nextera chemistry (Illumina Inc., San Diego, CA) and pair-end (2 × 101 bp) sequenced to average 2–3 M read pairs on HiSeq 2500 (Illumina Inc., San Diego, CA).

Analysis of obtained sequences was performed by core genome MLST (cgMLST) based on 2583 alleles in SeqSphere+ using SKESA assembler v.2.3.0 (Ridom GmbH, Münster, Germany). The cluster threshold was set to ≤6 genes difference between isolates [22].

### Statistical methods

Statistical analyses were performed using Statistica v.13.5.0.17 (Tibco software, Palo Alto, CA) and *p* < 0.05 was considered statistically significant. Categorical variables were compared using the χ^2^-test. Parametric and non-parametric continuous variables were compared using *t*-test and Mann Whitney *U* test, respectively. Mortality was analysed using Kaplan–Meier curves and Gehan’s Wilcoxon test. Logistic regression was used to detect bivariate associations among patient characteristics, RTs, and 30-day mortality.

### Ethics

The regional ethical board in Linköping (ref. 2014/108-31) approved the study.

## Results

### Ribotyping and clinical characteristics of CDI patients

Among the 366 examined isolates, 44 different *C. difficile* RTs were identified. RT046 was the most common, detected in 114 (31%) isolates, followed by RT012 (*n* = 44), RT014 (*n* = 30), RT001 (*n* = 15), RT002 (*n* = 8), RT011 (*n* = 7) and RT005 (*n* = 6). Clinical characteristics of the patients in the RT046 and comparison group are shown in Table 1. Among all patients with an HA infection (174/206; 84.5%) 96 (55.2%) suffered an infection caused by *C. difficile* RT046.

**Table 1.** Clinical characteristics of patients with primary *Clostridioides difficile* infection caused by PCR ribotype 046 compared to the six next most common ribotypes (comparison group).

Characteristic	Median (interquartile range), mean (SD) or no. (%) for patients infected with *C. difficile* of:		
	Ribotype 046 (*n* = 104)	Comparison group (*n* = 102)	*p*-value
Age in years	81.0 (74.5–86.0)	79.0 (71.0–86.0)	0.41
Female	51 (49.0)	64 (62.7)	0.05
CDI high-risk antimicrobials	93 (89.4)	85 (83.3)	0.20
Hospital associated *C. difficile* infection^a^	96 (92.3)	78 (78.0)	<0.01
Recurrence (≥1)	35 (33.7)	34 (33.3)	0.96
Multiple recurrence (≥2)	14 (13.5)	17 (16.6)	0.52
30-day mortality	21 (20.2)	8 (7.8)	0.01
One-year mortality	56 (53.8)	36 (35.3)	<0.01
Charlson comorbidity index	2.9 (±2.0)	2.7 (±1.9)	0.53
Intensive care unit	3 (2.9)	1 (1.0)	0.32

^a^ Two patients in the comparison group had primary infections that were defined as indeterminate.

*C. difficile* of RT027 was isolated from one patient and RT078 from two patients with no epidemiological linkage. No other hypervirulent ribotypes (RT017 and RT018) were found.

### Mortality and recurrence rate

**Figure 3.**
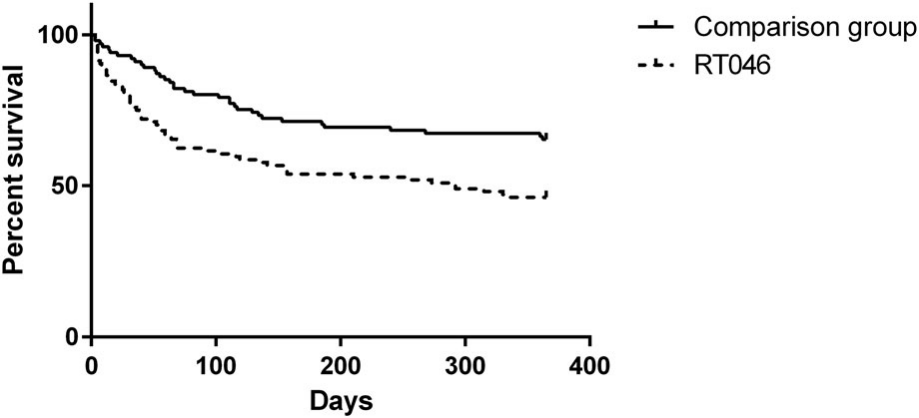
One-year survival rate after CDI among patients infected with *C. difficile* of RT046 (dashed line) compared to the comparison group (continuous line).

The overall mortality after 30 days was 14.1% and after 1 year 40.3%. Both 30-day and 1-year mortality rates were significantly higher among patients infected with *C. difficile* of RT046 compared to the comparison group (Table 1, Figure 3).

Logistic regression showed that patients infected with RT046 and patients with a Charlson comorbidity index ≥7 had a higher 30-day mortality rate, both when unadjusted and adjusted for RT, age and Charlson comorbidity index (Table 2).

**Table 2.** Risk factors for 30-day mortality among patients with *Clostridioides difficile* infection.

		Odds ratio (95% CI)		Odds ratio (95% CI)	
Characteristics	Number of patients who died within 30 days/total no. (%) of patients	Unadjusted	*p-*value	Adjusted	*p-*value
Ribotype					
Comparison group	8/102 (7.8)	ref		ref	
Ribotype 046	21/104 (20.2)	3.0 (1.3–7.1)	0.01	3.1 (1.3–7.5)	0.01
Age					
18–69	3/39 (7.7)	ref		ref	
70–79	6/57 (10.5)	1.4 (0.3–6.0)	0.79	not significant	
≥80	20/110 (18.2)	2.7 (0.7–9.5)	0.07	not significant	
Sex					
Female	15/115 (13.0)	ref			
Male	14/91 (15.3)	1.2 (0.6–2.7)	0.63		
Antibiotic prior to *C. difficile* infection					
Narrow	4/28 (14.3)	ref			
Broad	25/178 (14.0)	0.98 (0.3–3.1)	0.97		
Antibiotic treatment					
Metronidazole	19/153 (12.4)	ref			
Vancomycin (in combination or not with metronidazole)	5/32 (15.6)	1.3 (0.4–3.8)	0.89		
None	4/18 (22.2)	2.0 (0.6–6.8)	0.37		
Acquisition					
Community associated	3/30 (10.0)	ref			
Hospital associated	26/174 (14.9)	1.6 (0.4–5.6)	0.48		
Charlson comorbidity index					
0–3	16/145 (11.0)	ref		ref	
4–6	8/51 (15.7)	1.5 (0.6–3.7)	0.22	1.6 (0.6–4.2)	0.30
7–9	5/10 (50.0)	8.1 (2.1–30.9)	<0.01	8.2 (2.0–33.1)	<0.01

When comparing patients with HA primary infection in the two groups no statistically significant differences were found about age (*p* = 0.18) or Charlson comorbidity index (*p* =  0.84), but there was still a difference in mortality within 30 days and one year (*p* = 0.02 and *p* < 0.01, respectively).

The introduction of the Illumigene^TM^ C. difficile assay did neither reduce the 30-days mortality (*p* = 0.22) nor the recurrence rate (*p* = 0.65).

In total, 69 (33.5%) of the patients had one or more recurrences, and there was no difference in the recurrence rates between the two groups (Table 1). From the 43 patients where isolates from both the primary infection and the recurrence were available for typing, 40 patients had the same ribotype on both occasions.

Treatment with PPI did not affect recurrence rates. For patients treated with PPI, the recurrence rate was 36.7% (29 of 79 patients) compared to 31.0% (39 of 126 patients) for patients without treatment with PPI (*p* = 0.39).

### Inflammatory laboratory parameters associated with RT046 CDI cases

Patients with CDI caused by RT046 had a higher mean blood leukocyte count (16.9 × 10^9^ cells/l) than patients in the comparison group (13.4 × 10^9^ cells/l, *p* < 0.01).

Within the RT046 group, patients who died within 30 days after onset of infection had a higher mean blood leukocyte count (20.8 × 10^9^ cells/l) compared to those who survived more than a month (15.3 × 10^9^ cells/l, *p* < 0.01). This was not the case in the comparison group (*p* = 0.10).

The median CRP level was higher in patients with RT046 (118 mg/l, IQR = 70–204) compared to the comparison group (69 mg/l, IQR = 41–130, *p* < 0.01).

Lactate levels were not recorded for the majority of patients and creatinine was difficult to compare between patients due to differences in renal function. Therefore, these parameters were not analysed.

### Antibiotic treatment

CDI high-risk antimicrobials were given to 178 (86.4%) patients prior to CDI.

A majority of patients received metronidazole as treatment for their primary CDI (74.3%) (Table 3). The combination of vancomycin and metronidazole was more often used for patients with CDI of RT046 although the difference was not significant (*p* = 0.09).

**Table 3.** Antibiotic treatment for primary *Clostridioides difficile* infection caused by ribotype 046 compared to the six next most common ribotypes (comparison group).

Antibiotic treatment	No. (%) of patients infected with *C. difficile* of:		
	Ribotype 046 (*n* = 100)^a^	Comparison group (*n* = 85)^a^	*p-*value
Metronidazole	80 (80)	73 (85.9)	0.29
Vancomycin	5 (5)	6 (7)	0.56
Vancomycin and Metronidazole	15 (15)	6 (7)	0.09

^a^ Three patients with ribotype 046 and 15 in the comparison group did not receive any treatment. Data is missing for one patient in the ribotype 046 group and 2 in the comparison group.

There was no difference in time to treatment for the patients in the two groups (*p* = 0.68). The median time to treatment was shorter for patients whose samples were analysed with the Illumigene^TM^ *C. difficile* assay (0 days) compared to the Vidas CDAB assay (1 d, *p* < 0.01).

### Epidemiology and WGS data of the outbreak

The outbreak affected the entire hospital, and all wards had at least one case of CDI caused by RT046 during the study period. Figure 2 shows where patients were hospitalized during their CDIs caused by RT046 in 2010 and 2011. Wards with few patients with CDI (e.g. ICU, Table 1) are not shown. Most of the patients had been hospitalized in the same ward before symptoms (data not shown). The geriatric wards and medical wards A and B had new cases almost every month before the realization of the outbreak, but the numbers declined as soon as measures were taken to contain the outbreak. At the surgical wards, the number of new cases was constant over the entire period. Sporadic cases of CDI of RT046 were detected at medical ward C prior to the realization of the outbreak, whereas a cluster of cases was detected after measures to contain the outbreak were taken.

**Figure 4.**
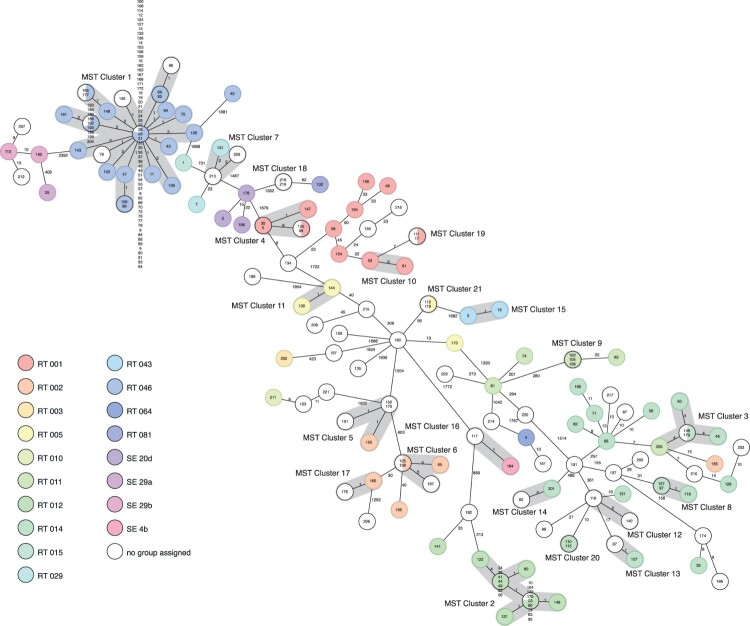
Ridom SeqSphere+ minimum spanning tree (MST) for 222 samples based on 2583 targets, pairwise ignoring missing values. Each colour of the circles represents a different ribotype. The size of the circles is not proportional to the number of isolates. The number on the lines shows the number of allelic differences. The length of the lines is not proportional to the allelic differences. Each number in the circles represents a single isolate and is according to the sampling date.

To further investigate the epidemiology, 246 isolates underwent WGS, generating sequences of high quality in 222 isolates. Based on a cluster threshold of ≤6 genes differences, 21 clusters (containing 2–88 isolates, in total 72% of the isolates clustered) were detected. The largest cluster (cluster 1, Figure 4) included isolates of the outbreak strain RT046, these isolates differed by 0–4 alleles and belonged to the same ST, namely ST35. One isolate of RT046 differed by 1881 genes from its nearest relative and was defined as another ST (Figure 4).

In the comparison group, the isolates differed by 0-several hundred alleles within each RT. Eighteen of 19 isolates of RT012 clustered (cluster 2, Figure 4). Isolates of RT002 were divided into three clusters (5, 6 and 17, Figure 4) and one singleton, belonging to two different STs. RT014 was seen in five clusters (3, 8, 13, 14 and 20, Figure 4) and eight singletons, belonging to five different STs. For the remaining RTs in the comparison group, all isolates of a single RT was the same WGS extracted ST, but divided into several clusters and singletons (Figure 4).

Of the isolates collected after the outbreak, nine were indistinguishable and clustered with isolates of the outbreak strain. Six of these came from patients hospitalized at medical ward C and three of these patients died within 30 days after onset of CDI. Another eight isolates belonged to the same STs as RT014, two to the same STs as RT002 and three to the ST corresponding to RT005. None belonged to the same ST as RT001or RT012.

Isolates from both the primary and recurrent infections were sequenced from 14 patients revealing 0–2 allelic differences between isolates within each patient.

Two isolates from the same patient and episode were characterized as two different RTs but WGS showed no gene differences.

## Discussion

The present study describes a large outbreak of *C. difficile* dominated by RT046, which was associated with a higher mortality compared to other RTs isolated during the same period. The increased mortality rate could not be explained by concomitant diseases, age, exposure to CDI high-risk antimicrobials, or choice of treatment for the primary infection.

High age, comorbidity, renal failure, and high leukocyte count have been considered risk factors for possible severe CDI [17,23]. Although we could not see any differences between the two groups according to age or comorbidity, patients with RT046 CDI had a higher mean blood leukocyte and median CRP count compared to patients in the comparison group, indicating that RT046 caused a more severe illness and by that more likely death.

Shortcomings in hygiene precautions most likely contributed to the outbreak, as has been described in other settings [24,25]. Before there was an awareness of the outbreak, patients were often transferred between wards after being exposed to *C. difficile* or while having diarrhoea. Sometimes the CDI patients were not isolated and the cleaning procedures around the patients were not optimal. The spread of RT046 might also have been facilitated by delayed diagnosis and readmission of patients with recurrences. This resulted in new patients being exposed to the bacteria. One of the measures to control the outbreak was to improve the hygiene standards by reinforcing adherence to standard precautions and cleaning procedures. As seen in Figure 2, simply recognizing the outbreak and making staff aware of the problem seem to have reduced the number of CDI RT046 cases at most medical and geriatric wards. This reduction in the incidence of RT046 might have been even greater since not all isolates before June 2011 were typed, and since Illumigene is a more sensitive diagnostic tool. However, at medical ward C, there was an increase in cases after the outbreak was recognized and this ward also had cases of the outbreak type after the outbreak was considered over. If this was due to lower compliance to hygiene guidelines, type of patients (lung – and gastrointestinal diseases) or the amount and types of antibiotics used is not known. When implementing the rapid diagnostic test in June 2011, time until diagnosis was reduced from 59 h to 2 hours [14], which presumably contributed to faster isolation of the patients. Isolation of CDI patients has been used as one of several methods to contain previous outbreaks [26,27]. The rapid diagnostic test also shortened time to treatment, which might have reduced transmission of the bacteria, despite having no effect on mortality. Further reduction of CDI cases was seen after the entire hospital was cleaned using sporicidal agents, also described by others [24,27]. Both a reduction of spores in the environment and the fact that departments were emptied from patients might have contributed to this reduction. After combating the outbreak, the number of new CDI cases per month has constantly been around 7.5/10,000 bed days [28] compared to 22 cases/10,000 bed days upon recognition of the outbreak. CDI with RT046 has not been discovered after 2013 in Högland Hospital.

To our knowledge, RT046 has only been described in two previous outbreaks [11,12]. Since the beginning of the twenty-first century several outbreaks due to *C. difficile* RT027, causing high morbidity and mortality, have been reported [2,3,26,29]. Outbreaks caused by other RTs *e.g.* 017 and 018 have also been reported [4,24,27,30,31]. Common to all these outbreak strains is multidrug resistance, *e.g.* to moxifloxacin [3,11,30] which we also have reported for RT046 [14]. Hence, multidrug resistance might also have contributed to the outbreak. Approximately one-third of *C. difficile* isolates are resistant to moxifloxacin in previous studies [32,33]. Extensive use of fluoroquinolones might contribute to the emergence of resistant *C. difficile* strains and resistance to fluoroquinolones seems to contribute to enhanced virulence among these RTs [4,5]. Specific restriction of fluoroquinolones was not used to contain this outbreak, as reported by others [34,35]. Other measures of antibiotic stewardship were implemented, to reduce unnecessary antibiotic prescriptions, as described previously [3]. Since the time of the outbreak, the prescription of moxifloxacin and other fluoroquinolones has decreased in our county (data not shown). Also, the proportion of multidrug-resistant isolates of *C. difficile* has decreased and the geographic clusters of specific *C. difficile* PCR ribotypes, including RT046, has disappeared in Sweden [21].

The proportion of CDI patients suffering from one or more recurrences (37%) in our study is larger compared to the recurrence rate (15–25%) in other studies [36,37]. At the time of the outbreak, metronidazole was commonly used in Sweden for the treatment of primary CDI and this might have contributed to the high rate of recurrences in our study. Musher *et al*. showed that only half of the patients treated with metronidazole were cured and almost one-third had a recurrence [38]. However, since the treatment regimens were similar in the two groups, the use of metronidazole probably did not contribute to the high mortality rate in the RT046 group. Treatment with fidaxomicin as well as prolonged oral vancomycin has shown to give fewer recurrences [36,39,40]. Fidaxomicin was, however, not available at the time of this outbreak and national recommendations have only recently been published, advocating a more frequent use of either fidaxomicin or vancomycin [23]. In our study, only a few patients received treatment with faecal microbiota transplantation, often after several recurrences. Shortcomings in cleaning and hygiene precautions might have contributed to the high recurrence rate as patients might have re-infected themselves through their contaminated hands and surrounding surfaces.

In a prospective multicentre study from Europe from 2012, RT 046 was not among the most common types but three of the types in our comparison group were among these (RT001, 002, and 014) [41]. This combined with the fact that RT046 clustered in WGS, and that most of the infections from RT046 were HA indicates that this strain was spread in the hospital environment.

Surveillance and typing of local CDI are important to discover clusters of the same type before it develops into larger outbreaks. The outbreak at Högland Hospital could have been detected earlier if a surveillance system and continuous typing would have been available during 2010. Today this routine has been implemented in Region Jönköping County; all samples positive for *C. difficile* are typed and if a cluster is discovered infection tracing begins and measures are put in place. Interpretation of DNA banding patterns, generated by PCR ribotyping, has previously been shown to be problematic [42], verified by data from our study. WGS is more suitable for outbreak investigation due to its objective data interpretation also shown by others [31].

The outbreak occurred almost 10 years ago, which is a limitation to this study. Still, it enlightens the need of surveillance of *C. difficile* in healthcare settings, and that other up to now known strain, such as RT027, 078, and 018, can cause outbreak. As this is a retrospective study, several clinical parameters, such as lactate and the frequency of diarrhoea, were hard to retrieve from medical records. The cause of death was also hard to evaluate retrospectively. All isolates were not stored and therefore not possible to analyse. It was not possible to trace exactly in which rooms patients were hospitalized. Another limitation in our study is the use of different strain typing methods. Also, due to limited resources, we were not able to ribotype all the positive samples during the outbreak. In an outbreak situation, like this, several measures must be in place simultaneously to reduce suffering and even the death of patients. To evaluate each effort before putting the next into place would be ideal for research but not ethical.

In conclusion, *C. difficile* of RT046 seems to be highly pathogenic and able to spread effectively in a hospital environment. Since there were no differences in concomitant disease, age, or antibiotic treatment between the two groups, this indicates that RT046 causes higher mortality compared to other RTs. Several factors, *e.g.* monthly evaluations of new CDIs, sporicidal cleaning procedures, implementation of a rapid diagnostic test, and antibiotic stewardship contributed to limit the outbreak. Full control was first achieved when this bundle of measures was implemented, leading to an awareness of the outbreak among physicians and health care workers, which we believe, is a prerequisite to contain an outbreak.
